# Women’s knowledge and attitudes towards cervical cancer prevention: a cross sectional study in Eastern Uganda

**DOI:** 10.1186/s12905-017-0365-3

**Published:** 2017-01-31

**Authors:** Trasias Mukama, Rawlance Ndejjo, Angele Musabyimana, Abdullah Ali Halage, David Musoke

**Affiliations:** 1 Department of Disease Control and Environmental Health, School of Public Health, College of Health Sciences, Makerere University, P.O Box 7072, Kampala, Uganda; 2 Department of Community Health, School of Public Health, College of Medicine and Health Sciences, University of Rwanda, Kigali, Rwanda

**Keywords:** Attitudes, Cervical cancer, Knowledge, Prevention, Rural, Screening, Uganda

## Abstract

**Background:**

Cervical cancer is a leading cause of morbidity and mortality among women in Uganda, often due to late disease diagnosis. Early screening for the cancer has been shown to be the most effective measure against the disease. Studies conducted elsewhere have reported the lack of awareness and negative attitudes towards cervical cancer as barriers to early screening. This study assessed the knowledge and attitudes of Ugandan women about cervical cancer prevention with the aim of informing prevention and control interventions.

**Methods:**

This study was conducted in Bugiri and Mayuge districts in eastern Uganda. It was a cross-sectional community based survey and collected data by means of a questionnaire. A total of 900 women aged 25–49 years participated in the study. Women’s knowledge and attitudes towards cervical cancer prevention were assessed and scored. Data were analysed using STATA 12.0 software. Bivariate and multivariate analyses were carried out to establish the relationship between knowledge levels and demographic characteristics.

**Results:**

Most (794; 88.2%) of the respondents had heard about cervical cancer, the majority (557; 70.2%) having received information from radio and 120 (15.1%) from health facilities. Most women (562; 62.4%) knew at least one preventive measure and (743; 82.6%) at least one symptom or sign of the disease. The majority (684; 76.0%) of respondents perceived themselves to be at risk of cervical cancer, a disease most (852; 94.6%) thought to be very severe. Living in peri-urban areas (AOR = 1.62, 95% CI: 1.15 – 2.28), urban areas (AOR = 3.64, 95% CI: 2.14 – 6.19), having a higher monthly income (AOR = 0.50, 95% CI: 0.37 – 0.68) and having had an HIV test (AOR = 1.99, 95% CI: 1.34–2.96) were associated with level of knowledge about cervical cancer prevention.

**Conclusion:**

Although general knowledge about cervical cancer prevention was relatively high among women, and attitudes mostly encouraging, specific knowledge about screening was low. There were also undesirable perceptions and beliefs regarding cervical cancer among respondents. There is therefore need for more education campaigns to bridge identified knowledge gaps, and scale up of cervical cancer screening services to all women to increase service uptake.

**Electronic supplementary material:**

The online version of this article (doi:10.1186/s12905-017-0365-3) contains supplementary material, which is available to authorized users.

## Background

Cervical cancer is the second most common cancer among women in the developing world and is responsible for 230,200 deaths and 444,500 cases annually [1–3]. It is a major cause of morbidity and mortality in resource-poor settings where access to cervical cancer screening and vaccination is limited [4, 5]. Over 80% of cervical cancers in sub-Saharan Africa are detected in late stages, predominantly due to lack of information about the disease and lack of screening services [4, 5]. Consequently, women with cervical cancer in this region are not identified until they are at an advanced stage of disease which is associated with low survival rates [6]. East Africa has the highest age-standardised incidence rates for cervical cancer at 42.7 per 100,000 women per year [2]. In Uganda, an estimated 33.6% of women in the general population harbour human papillomavirus, a necessary cause of cervical cancer, and 44 per 100,000 women develop the disease every year [7]. With 3,915 women diagnosed with cervical cancer annually, Uganda ranks 14^th^ among countries with the highest incidence rates [7]. Amongst Ugandan women of reproductive age, the risk of developing cancer is high.

Although Uganda lacks a cervical cancer screening policy, the ministry of health’s strategic plan for cervical cancer prevention and control aimed to reach 90% of Ugandans with information education and communication materials about cervical cancer and to screen up to 80% of eligible women aged 25–49 years [8]. These efforts led to establishment of cervical cancer screening centres in national and regional referral hospitals, private-not-for-profit and private-for-profit hospitals. This notwithstanding, access to cervical cancer screening services remains limited especially for rural women. The success of a cervical cancer screening programme depends on access and uptake, the quality of screening tests, the adequacy of follow-up, and diagnosis and treatment of pre-cancerous and cancerous lesions detected.

Available evidence so far suggests that cervical cancer services have not been optimally utilised in Uganda. For instance, a recent study conducted in central Uganda found that only 7% of women had ever been screened for cervical cancer [9] while another in Eastern Uganda reported 4.8% [10]. Several factors, both individual and attitudinal influence women’s decision to undergo cervical cancer screening [9]. Studies show that having sufficient knowledge about cervical cancer and screening programmes increase acceptance, and uptake of available screening services [11–13]. Although knowledge plays a critical role in influencing a woman’s decision to screen, some women, nevertheless do not undergo screening. For example, studies conducted among health workers, who are expected to be knowledgeable, have also found low screening uptake rates [14, 15]. Therefore, women’s attitudes towards cervical cancer and screening are equally important. Attitude regarding perceived risk, screening methods used, perceived pain during screening have been suggested to influence decisions to undergo the procedure [9, 12, 15]. Data on the knowledge and attitudes of women towards cervical cancer prevention in eastern Uganda is limited. This study determined women’s knowledge and attitudes towards cervical cancer prevention as determinants for utilization of preventive services.

## Methods

### Study design and area

This was a cross-sectional study conducted using a community based questionnaire survey which collected quantitative data. The questionnaire was administered by research assistants to women who were found in their homes. The study was conducted in Bugiri and Mayuge districts in eastern Uganda. The districts are approximately 150 km from Kampala, the capital city of Uganda. Bugiri and Mayuge districts are predominantly rural with most residents involved in subsistence farming with emphasis on crop growing as the main economic activity. The districts are located along the shores of Lake Victoria and communities that border with the lake are involved in fishing. Other residents who live in small towns and trading centres within the districts are involved in small scale businesses. The majority of people in the districts reside in roofed mud and wattle houses. Bugiri is composed of nine sub-counties while Mayuge has seven sub-counties. Both districts have an estimated combined population of 856,152 people of whom 51.4% are females [16] and a combined area of 10,372 km square. Cervical cancer screening services in the two districts are provided by Bugiri district hospital which also serves other neighbouring districts. The district hospital provides intermittent cervical cancer screening services and treatment of those diagnosed with the disease. Two private health facilities, both located in Bugiri town, provide cervical cancer screening in Bugiri district and one private facility serves Mayuge district.

### Study population and eligibility

The study involved females aged 25 to 49 years in the selected districts who had lived in the area for more than six months. The sampling units were households and only one participant was selected per sampled household.

### Sampling procedure

A multi-stage sampling technique was used: five sub counties were randomly selected from each district. Five villages were then selected from each sub county using simple random sampling to obtain 25 study villages in each district. In order to select the households, systematic random sampling was used where the interval for selection of the households was determined by dividing the approximate number of households in a given village by the required number of respondents from each village. Lists of sub-counties and villages were obtained from district officials while village local leaders provided the estimates of numbers of households in their villages. Within households, simple random sampling was used to select a respondent whenever more than one eligible woman were present at the time of data collection.

### Data collection

Data was collected using a questionnaire (see Additional file 1) that captured information on knowledge and attitudes of participants on cervical cancer prevention. The questionnaire, which was translated to *Lusoga*—the main language used in the study area—and back translated to English with any discrepancies addressed was pretested among a group similar to the study respondents. The survey questionnaire had five sections. The first section included questions on the participants’ demographic characteristics such as age, highest level of education attained, marital status, area of residence, and number of children, previous health seeking behaviours, and use and methods of contraception. The second section had questions on awareness and sources of information about cervical cancer prevention. The third section included ten questions that assessed the respondents’ specific knowledge about cervical cancer prevention measures, symptoms and screening methods. This section also assessed women’s knowledge of recommended age for cervical cancer vaccination, screening and the frequency of screening. Some questions required Yes/No/I don’t know responses while others required the participant to mention responses. The fourth section comprised of a list of ten questions on risk factors which comprised both factual and common myths about cervical cancer. The risk factors included multiple sexual partnerships, smoking, use of contraception, heredity, previous exposure to sexually transmitted diseases and early sex onset. A knowledge score was generated for the third and fourth sections with 1 point given for one correct response for a maximum possible 20 points. The last section included questions on attitudes and required respondents to state their level of agreement with statements about cervical cancer on a 5-point Likert scale from 1 (strongly disagree) to 5 (strongly agree). This section had ten statements that assessed women’s perception of risk, severity of cervical cancer, perceived self-efficacy and the importance of cervical cancer screening.

### Data entry and analysis

Data were entered and cleaned in Epidata 3.02 (EpiData Association, Denmark) and transferred to Stata SE statistical software (version 12.0 College Station, Texas) for analysis. Descriptive statistics were conducted to characterize the participants and provide frequencies on individual questions and attitudes. Bivariate analysis was conducted to determine the association between socio-demographic characteristics and knowledge about cervical cancer prevention. To obtain a binary outcome of knowledge, the mean knowledge score was determined and women who had scores above the mean were considered to be more knowledgeable while those who scored below were considered to have less knowledge. This formed the outcome variable which was coded as 1 for high knowledge and 0 for low knowledge and run against the socio-demographic characteristics to identify the predictors for high knowledge among the respondents. A multivariable model that adjusted for confounding was developed. Variables were added in the model based on a statistical significance of ≤ 0.15 at bivariate level and biological plausibility. Odds ratios and 95% confidence intervals were used as measures of association.

## Results

A total of 900 women responded to the questionnaire. The mean age of respondents was 32.9 years (Standard Deviation [SD] = 6.7) and most (530; 58.9%) had completed primary education. The majority of respondents were married (767; 85.2%), engaged in farming (499; 55.4%) and resided in rural areas (610; 67.8%) (Table 1).

**Table 1:** Socio-demographic characteristics of study participants, *N* = 900

Characteristic	Categories	Frequency (%)
District	Bugiri	452 (50.2)
	Mayuge	448 (49.8)
Residence	Rural	610 (67.8)
	Semi-urban	195 (21.7)
	Urban	95 (10.5)
Age	Mean ± SD	32.9 ± 6.7
	25-29	374 (41.6)
	30-39	329 (36.5)
	40-49	197 (21.9)
Religion	Christian	518 (57.6)
	Muslim	382 (42.4)
Education	None	142 (15.8)
	Completed primary	530 (58.9)
	Completed secondary (ordinary level)	228 (25.3)
Marital status	Married	767 (85.2)
	Not married	133 (14.8)
Occupation	Farming/Agriculture	502 (55.8)
	Trade/Business	215 (23.9)
	Housewife	183 (20.3)
Parity	Mean ± SD	5.0 ± 2.7
	0-3	270 (30.0)
	4-6	389 (43.2)
	7+	241 (26.8)
Monthly household income (US dollars)	≤40	622 (69.1)
	>40	378 (30.9)
Use modern family planning method	Yes	583 (64.8)
	No	317 (35.2)
Ever had an HIV test	Yes	756 (84.0)
	No	144 (16.0)

### Knowledge about cervical cancer and risk factors

Almost all women (898; 99.8%) had heard about cancer and the majority (794; 88.2%) had heard about cervical cancer. The main sources of information about cervical cancer were radio (557; 70.2%), health centres (120; 15.1%) and networks of friends and family members (104; 13.1%). The majority (854; 94.9%) of respondents stated that early detection of cervical cancer was helpful in its treatment while 671 (74.6%) knew that the disease was curable if detected early. Among the respondents, 625 (69.4%) said that cervical cancer could be prevented with 562 (62.4%) correctly stating at least one preventive measure of the disease. Only 7 (0.01%) respondents knew the recommended frequency for cervical cancer screening and 743 (82.6%) stated at least one symptom of the cancer (Table 2). Overall 499 (55.4%) of the women had high knowledge about cervical cancer and its risk factors.

**Table 2:** Knowledge about cervical cancer and its preventive and control measures, *N* = 900

Prompt	Frequency (%)
1. Early detection of cervical cancer is helpful in its treatment	854 (94.9)
2. Cervical cancer is curable if detected early	671 (74.6)
3. Someone can be vaccinated against cervical cancer	578 (64.2)
4. Knew the recommended age for girls to undergo vaccination against cervical cancer^a^	196 (21.8)
5. Knew the age when a woman should start undergoing cervical cancer screening^b^	37 (4.1)
6. Knew the frequency for cervical cancer screening^b^	7 (0.01)
7. Cervical cancer can be prevented	625 (69.4)
8. Knew at least one preventive measure for cervical cancer	562 (62.4)
9. Knew at least one symptom of cervical cancer	743 (82.6)
10. Knew at least one test used to screen for cervical cancer	411 (45.7)

^a^WHO recommends vaccination for girls aged 9–15 years

^b^WHO recommends starting screening for women aged 30 years and continuing at three-year intervals

Knowledge about the risk factors for cervical cancer was high with most (706; 78.4%) respondents stating that having multiple sexual partners, being infected with the human papilloma virus (HPV) (760; 88.4%) and starting to have sexual intercourse at a young age (665; 73.9%) increased a woman’s risk of developing cervical cancer. Most women (713; 79.2%) also thought that using contraceptives for a long time increased one’s risk of developing the cancer (Table 3).

**Table 3:** Knowledge about risk factors for developing cervical cancer, *N* = 900

Factor	Frequency (%)
1. Smoking	583 (64.8)
2. Many sexual partners	706 (78.4)
3. Human papilloma virus	760 (84.4)
4. Sexually transmitted diseases	736 (81.8)
5. Human immunodeficiency syndrome	672 (74.7)
6. Early onset of sexual activity	665 (73.9)
7. Family history of cervical cancer	442 (49.1)
8. Uncircumcised male partner	582 (64.7)
9. Use of contraceptive pills for a long time	713 (79.2)
10. Living with a cervical cancer patient	332 (36.9)

**Fig. 1:**
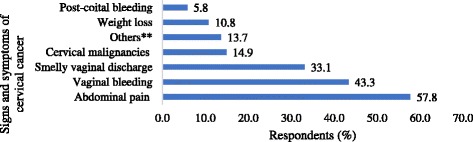
Knowledge about signs and symptoms of cervical cancer, *N* = 900. Others** include; vaginal itching, backache, vaginal sores and painful sex

The most known measures to prevent cervical cancer among women were early screening (414; 46%) and vaccination (300; 33.3%). Others (83; 9.2%) thought that measures such as safe male circumcision, using condoms and avoiding multiple sexual relationships could prevent cervical cancer. Only three (0.3%) thought that nothing could be done to prevent cervical cancer while 154 (17.1%) did not know any method of preventing the disease. Regarding knowledge about signs and symptoms of cervical cancer, abdominal pain (520; 57.8%), vaginal bleeding (390; 43.3%) and smelly vaginal discharge (298; 33.1%) were the commonly known (Fig. 1). More than half of respondents (489; 54.3%) did not know any methods used for screening for cervical cancer. Other respondents knew liquid-based cytological screening (22.8%), HPV test (21.2%) and the Pap smear test 117 (13%) as methods for cervical cancer screening.

### Predictors of higher knowledge about cervical cancer prevention among women

At bivariate analysis, household income, area of residence and having ever had an HIV test were significantly associated with knowledge among respondents. When potential confounders were adjusted for, women who lived in urban and semi-urban areas were four times (adjusted odds ratio (AOR) = 3.64, 95% confidence interval (CI): 2.14 – 6.19) and two times (AOR = 1.62, 95% CI: 1.15 – 2.28) more likely to have high knowledge about cervical cancer than their rural counterparts respectively. Respondents who earned more than 40 US dollars per month were 50% less likely to be knowledgeable (AOR = 0.50, 95% CI: 0.37 – 0.68) while those who had ever had an HIV test were two times (AOR = 1.99, 95% CI: 1.34 – 2.96) more likely to be knowledgeable about cervical cancer compared to their counterparts (Table 4).

**Table 4:** Crude and adjusted odds ratios for predictors of knowledge about cervical cancer and risk factors

Predictor variables	Categories	COR (95%CI)	*p*-value	AOR* (95%CI)	*p*-value
Age	25–29	1		1	
	30–39	1.03 (0.76–1.39)	0.854	1.17 (0.82–1.67)	0.381
	40–49	0.84 (0.59–1.18)	0.319	1.02 (0.66–1.56)	0.944
Education	No education	1		1	
	Completed primary	1.32 (0.91–1.91)	0.141	1.16 (0.78–1.73)	0.469
	Completed ordinary secondary	1.39 (0.91–2.11)	0.126	1.01 (0.63–1.62)	0.954
Religion	Christian	1			
	Muslim	0.84 (0.64–1.09)	0.184		
Residence	Rural	1		1	
	Semi-urban	**1.73 (1.24–2.41)**	**<0.001**	**1.62 (1.15–2.28)**	**0.006**
	Urban	**3.36 (2.03–5.56)**	**<0.001**	**3.64 (2.14–6.19)**	**<0.001**
Occupation	Farming	1			
	Business/salaried work	0.77 (0.56–1.07)	0.196		
	Housewife	0.75 (0.54–1.06)	0.165		
Marital status	Married	1			
	Single	0.71 (0.49–1.02)	0.066		
Parity	0–3	1			
	4–6	0.82 (0.60–1.23)	0.226		
	7+	0.77 (0.54–1.09)	0.136		
Monthly household income	Less than 40 US Dollars	1		1	
	More 40 US Dollars	**0.57 (0.42–0.75)**	**<0.001**	**0.50 (0.37–0.68)**	**<0.001**
Use modern family planning method	No	1			
	Yes	1.14 (0.87–1.50)	0.343		
Ever had an HIV test	No	1		1	
	Yes	**2.65 (1.83–3.84)**	**<0.001**	**1.99 (1.34–2.96)**	**0.001**

AOR* - Mutually adjusted for: age, education, urban–rural residence, monthly household income and having previously had an HIV test

*AOR* adjusted odds ratio, *COR* crude odds ratio

### Attitudes towards cervical cancer prevention

Most women (852; 94.7%) thought that cervical cancer was a severe disease and the majority (684; 76.0%) believed that they were at risk of developing it. The majority (850; 94.4%) of respondents believed that cervical cancer screening was important and 706 (78.4%) knew that the chances of curing the disease were higher if diagnosed early. A significant number (747; 83.0%) believed that cervical cancer was symptomatic and therefore infected women would have signs and symptoms of the disease. Also, most respondents (556; 61.8%) believed that nothing could be done once someone is diagnosed with cervical cancer (Table 5).

**Table 5:** Attitudes of women towards cervical cancer prevention

Statement	Strongly disagree (%)	Disagree (%)	Neutral (%)	Agree (%)	Strongly agree (%)
1. Cervical cancer is a very severe disease	39 (4.3)	3 (0.3)	6 (0.7)	269 (29.9)	583 (64.9)
2. I am at risk of getting cervical cancer	38 (4.2)	50 (5.6)	128 (14.2)	476 (52.9)	208 (23.1)
3. Cervical cancer screening is important	32 (3.6)	6 (0.7)	12 (1.3)	428 (47.6)	422 (46.9)
4. Only women who are sexually active need cervical cancer screening	107 (11.9)	299 (33.2)	95 (55.7)	277 (30.8)	122 (13.6)
5. Women who have had sexually transmitted diseases are more likely to get cervical cancer	67 (7.4)	133 (14.8)	82 (9.1)	446 (49.6)	172 (19.1)
6. Once cervical cancer has been diagnosed, something can be done about it	127 (14.1)	429 (47.7)	92 (10.2)	164 (18.2)	88 (9.8)
7. Chances of curing cervical cancer are better when the disease is discovered at an early stage	52 (5.8)	76 (8.4)	66 (7.3)	490 (54.4)	216 (24.0)
8. Cervical cancer is not a death sentence for most people	144 (16.0)	305 (33.9)	102 (11.3)	248 (27.6)	101 (11.2)
9. There is much a woman can do to reduce her chances of getting cervical cancer	47 (5.2)	261 (29.0)	149 (16.6)	320 (35.6)	123 (13.7)
10. Women who have cervical cancer will have some signs to show it	40 (4.4)	37 (4.1)	76 (8.4)	466 (51.8)	281 (31.2)

## Discussion

This study found that most women were knowledgeable about cervical cancer symptoms, prevention measures and risk-factors. This is consistent with findings from a similar study conducted in northern Uganda [17]. This high awareness indicates that women may be in position to recognize cervical cancer basing on its symptoms and seek medical attention. Also, when women are aware of the causes and risk factors of cervical cancer and perceive themselves to be at risk, they are more likely to take up measures to prevent the acquisition of human papilloma virus hence avoid developing the disease. Indeed, previous studies have showed that awareness of cervical cancer symptoms and prevention measures, and perception of being at risk of the disease were associated with intention to go for screening and thus its early detection [12, 18–20]. However, studies conducted among health workers in Uganda and Nigeria, who are expected to be knowledgeable, found low screening rates [14, 21]. Cervical cancer awareness campaigns should focus on increasing knowledge of signs and symptoms and risk perception of the disease to encourage screening and facilitate its early detection.

This study found knowledge gaps that might inhibit women from undergoing cervical cancer screening. For instance, most women did not know the recommended age to start screening and even fewer knew the recommended frequency of screening for the disease. In addition, the belief that nothing can be done once one is diagnosed with cervical cancer was common and might hinder women from seeking screening services for fear of a positive diagnosis. Other studies have also reported gaps in knowledge among women in various settings [14, 21–24]. In this study, radios, health workers and networks of significant others were the principal sources of information for most women. Also, previous Ugandan studies have showed that significant others such as paternal aunts are important sources of reproductive health information [23] and that men play an important role in influencing women’s decision to go for screening [9]. Likewise, health workers have been shown to be an important source of such information [13, 25]. Education campaigns aiming at providing comprehensive knowledge about the disease should utilise radios and health workers and should focus on addressing identified knowledge gaps.

Having previously tested for HIV/AIDS and residing in an urban or peri-urban area were associated with high cervical cancer knowledge among women. This is possibly because people who have tested for HIV may have good health seeking behaviours and have had interaction with health facilities, a key source of cervical cancer related information. Also, town residents are in close proximity to these health facilities that provide cervical cancer services. These findings suggest that integrating HIV counselling and testing services with cervical cancer services would enhance awareness about the disease among women. A study conducted in Uganda showed that such integration, although might result into longer waiting hours at the health facilities, is to a large extent manageable by both health workers and women [26]. In our study, women who belonged to the lower socio economic category were more knowledgeable about cervical cancer prevention compared to those from the higher status. This finding is surprising and seemingly counterintuitive. However, it could reflect service utilisation trends in rural areas whereby long waiting hours and poor quality of services at health facilities may act as disincentives and hinder working women (higher income women) from seeking care yet these health facilities could be the major source of information on cervical cancer in rural settings. In a study by Jia conducted in China, women who had lower incomes had higher willingness to screen compared to their other counterparts [19] while a Botswanan study found that previous cervical cancer screenings was high among women of higher incomes [27]. Cervical cancer screening services should also extend to rural areas since a woman’s awareness of location of a service point is associated with acceptance and uptake of screening [10–12] and should also target women in the higher economic stratum.

Most women showed a positive attitude towards cervical cancer screening. For instance, most women thought that early disease diagnosis was helpful in disease treatment and that they were at risk of getting cervical cancer, which they believed was a severe disease. Women’s perception of being at risk of cervical cancer was earlier found to be associated with their intention to go for screening services [9]. Since earlier studies have showed that attitudinal factors such as not feeling susceptible to cervical cancer and having limited knowledge about the disease affect uptake of services [12, 14], the fact that many women were generally knowledgeable about cervical cancer and had a positive attitude presents an opportunity for cervical screening programmes. Therefore, it is likely that if more screening opportunities are presented to women, many will screen for the disease and take up preventive measures. It is also worth noting that in some studies, even when the opportunity to screen was provided to women, they reported other barriers such as fear of a positive cervical cancer diagnosis, and other fears related to the screening procedures and vaginal examinations [12, 28] and therefore programs should be designed to provide treatment for those diagnosed with cancer. Cervical cancer awareness and prevention programmes should continually seek to influence women’s perceptions about cervical cancer and screening.

We also found perceptions that might negatively impact providing care to cervical cancer patients and affect public health interventions. For example, a significant number believed that cervical cancer patients could transmit the disease and most women thought that long-term use of contraceptives could cause cervical cancer. The belief that use of contraceptives leads to cervical cancer has been documented in studies carried out in other regions of Uganda [17, 24, 29]. This perception could be due to the similarity between the side effects of some of the contraceptive methods and the gynecological signs of cervical cancer such as longer periods of menstrual bleeding. This perception might hinder women from utilising contraceptives, reducing effectiveness of measures towards reducing fertility rates, which are among the highest in the world.

### Strengths and limitations

This was a community based study that involved women eligible for cervical cancer screening in eastern Uganda. The study provides insights into the knowledge and attitudes of women in rural areas towards cervical cancer prevention. This information is important for designing appropriate interventions to increase cervical cancer awareness and screening in such areas where it has been reported as very low by previous studies, whilst acting as a benchmark for evaluating such interventions. A limitation of this study is the lack of a standardised knowledge assessment questionnaire which limits the comparability of the findings across studies carried out in the region.

## Conclusion

This study found relatively high knowledge about general cervical cancer prevention but specific knowledge about screening was very low. There were also positive attitudes towards cervical cancer prevention. Since high knowledge and positive attitudes themselves are not enough to ensure uptake of screening services, there is need to scale up such cervical cancer screening services so that more women can access them irrespective of where they reside. There is also need for more awareness campaigns to provide comprehensive information about cervical cancer screening to women in all areas and dispel any negative beliefs and perceptions.

## Additional file

Additional file 1: Women’s knowledge and attitudes towards cervical cancer prevention. Description of file: Study questionnaire. (PDF 456 kb)

## Abbreviations

AIDS: Acquired immune deficiency syndrome
AOR: Adjusted odds ratio
CI: Confidence interval
COR: Crude odds ratio
HIV: Human immunodeficiency virus
HPV: Human papilloma virus
SD: Standard deviation
