# Supplementary material for: Women’s knowledge and attitudes towards cervical cancer prevention: a cross sectional study in Eastern Uganda
Source: BMC Womens Health. 2017 Jan 31;17:9. doi: 10.1186/s12905-017-0365-3 (PMC5282746; doi:10.1186/s12905-017-0365-3)
Supplement: Additional file 1: — Women’s knowledge and attitudes towards cervical cancer prevention. Description of file: Study questionnaire. (PDF 456 kb) [file 12905_2017_365_MOESM1_ESM.pdf]

Women's knowledge and attitudes towards cervical cancer prevention: A cross sectional study in Eastern Uganda

Study questionnaire
Version: 1.2; August 26th, 2014

Section I: Interview parameters

1.1 Questionnaire number: | | | | 1.2 Date of interview: |__|__/0|9/2014

1.3 Name of Interviewer: ____________________

1.4 District: ________________________________

1.5 Sub County: ____________________________ 1.6 Village: ________________________________

1.7 Start time: ___________________________ 1.8 Name of Data Editor: _______________________

ELIGIBILITY: RESPONDENT SHOULD BE AGED BETWEEN 25 TO 49 YEARS AND SHOULD HAVE SPENT SIX (6) OR MORE MONTHS IN THE DISTRICT

Write 99 in case respondent refuses to answer

Section II: Sources of information about cervical cancer

s/n	Question	Response	For editor
2.1	Have you ever **heard** about cancer?	1. Yes<br>2. No	
2.2	Have you ever **heard** about cervical cancer?<br>**NO ⟶ Next section**	1. Yes<br>2. No	
2.3	Where did you hear about cervical cancer from?<br><br>***(PROBE and Tick all that apply)***	1. Radio<br>2. Television<br>3. Posters<br>4. Health facility<br>5. Friend/family member<br>6. Newspapers / magazines<br>7. School/learning institution<br>8. Other________________________<br>9. Don't know or remember	

Section III: Knowledge about cervical cancer and screening

3.1	Do you think that early detection of cervical cancer is helpful?	1. Yes<br>2. No<br>3. Don't know	
3.2	Do you think that cervical cancer is curable if detected early?	1. Yes<br>2. No<br>3. Don't know	
3.3	Can someone be vaccinated against cervical cancer?<br>**NO⟶ 3.5**	1. Yes<br>2. No<br>3. Don't know	
3.4	What is the recommended age for someone to undergo the vaccination?	1. ________________ years<br>2. Don't know	
3.5	At what age should a woman start screening for cervical cancer?	1. ________________ years<br>2. Don't know	
3.6	How frequently should someone be screened for cervical cancer?	1. Every ________________ Months (write 00 if less than 1 month)<br>2. Don't know	
3.7	Do you think cervical cancer can be prevented?<br>**NO⟶ 3.9**	1. Yes<br>2. No<br>3. Don't know	
3.8	What cervical cancer preventive measures do you know?<br>***(PROBE and TICK all that apply)***	1. Early screening<br>2. Avoiding smoking<br>3. Vaccination<br>4. Nothing can be done<br>5. Others ____________________________<br>6. Don't know	
3.9	What are some of the symptoms of cervical cancer that you know?<br><br>***(PROBE and TICK all that apply)***	1. Vaginal bleeding<br>2. Post-coital bleeding<br>3. Weight loss<br>4. Foul smelling vaginal discharge<br>5. Abdominal pain<br>6. Malignancies<br>7. Other ____________________________<br>8. Is asymptomatic	
3.10	Which tests can be carried out to detect cervical cancer?<br>***(PROBE and TICK all that apply)***	1. Pap smear test<br>2. Cytological screening<br>3. HPV test<br>4. Don't know	

**3.11**	**Total score of knowledge about cervical cancer and screening**		**10**

Section IV: Knowledge about risk factors for cervical cancer

Interviewer to read each of the factors to the respondents and then tick the appropriate response provided

Which of the following are risk factors for cervical cancer?

s/n	Factor	Yes	No	Don´t know
4.1	Smoking			
4.2	Many sexual partners			
4.3	Human papilloma virus (HPV)			
4.4	Sexually transmitted diseases			
4.5	Human immunodeficiency virus (HIV)			
4.6	Early onset of sexual activity			
4.7	Family history of cervical cancer			
4.8	Uncircumcised male partner			
4.9	Use of contraceptive pills for a long time			
4.10	Living with a cervical cancer patient			

**4.11**	**Score of knowledge on risk factors**		**10**

**4.12**	**Total score of knowledge about cervical cancer and risk factors**		**20**

Section V: Attitudes towards cervical cancer and screening

Interviewer to read each of the factors to the respondents and then record the numbers corresponding with the response given

To what extent do you agree or disagree with the following statements?

s/n	Statement	Strongly disagree [1]	Disagree [2]	Neutral [3]	Agree [4]	Strongly agree [5]
5.1	Cervical cancer is a very severe disease					
5.2	I am at risk of getting cervical cancer					
5.3	Cervical cancer screening is important					
5.4	Only women who are sexually active need cervical cancer screening					
5.5	Women who have had sexually transmitted diseases are more likely to get cervical cancer					
5.6	Once cervical cancer has been found, there is not much that can be done about it					
5.7	Chances of curing cervical cancer are better when the disease is discovered at an early stage					
5.8	Cervical cancer is a death sentence for most people					
5.9	There is little a woman can do to reduce her chances of getting cervical cancer					
5.10	Women who have cervical cancer will have some kind of symptoms showing it					
5.11	Cervical cancer runs in families					
5.12	Women only need cervical cancer screening tests during child bearing years.					
5.13	I would vaccinate myself against cervical cancer					
5.14	I Would allow my children to be vaccinated against cervical cancer					
5.15	My family would approve of children being vaccinated against cervical cancer					

Section VI: Respondent characteristics

s/n	Question	Coding categories	For editor
6.1	How old are you?<br>***(Record age in completed years)***	______________________________	
6.2	What is your religion	1. Catholic<br>2. Protestant<br>3. Muslim<br>4. Pentecostal<br>5. SDA<br>6. Other _________________________	
6.3	What is the highest level of education you attained?	1. None<br>2. Primary (P1 to P7)<br>3. O level (S1 to S4)<br>4. A level (S5 to S6)<br>5. Tertiary education/University	
6.4	What is your tribe?	1. Musoga<br>2. Muganda<br>3. Other ________________________	
6.5	What is your marital status?<br><br>**1, 3 or 4 ⟶ 6.7**	1. Single<br>2. Married<br>3. Widowed<br>4. Divorced/Separated	
6.6	Are you in a monogamous or polygamous relationship?	1. Monogamous<br>2. Polygamous	
6.7	What is your occupation?	1. Agriculture / Farming<br>2. Business / Trade<br>3. Housewife<br>4. Civil servant<br>5. Other _________________________	
6.8	Are you the head of your household?<br>**YES ⟶ 6.10**	1. Yes<br>2. No	
6.9	What is your relationship with the head of the household?	1. Wife<br>2. Parent<br>3. Sister<br>4. Daughter<br>5. Other Relative<br>6. Not related	
6.10	How many deliveries have you had?	____________________________	
6.11	How many people stay in this household on a regular basis?	____________________________	
6.12	What is your average household monthly income?	________________ Uganda Shillings	
6.13	How long have you lived in this district?	1. Less than 1 year<br>2. Between 1 to 5 years<br>3. More than 5 years	

		4. All my life<br>5. Don't know / remember	
6.14	Where do you reside?<br>***(Interviewer to ask and determine)***	1. Rural area<br>2. Semi-urban area<br>3. Urban area	
6.15	Have you ever used any method to delay or avoid getting pregnant?<br>**NO ⟶ 6.17**	1. Yes<br>2. No	
6.16	Which methods have you used?<br><br>***(Tick all that apply)***	1. Female sterilization<br>2. Pill<br>3. IUDs<br>4. Injectables<br>5. Inplants<br>6. Condom<br>7. Diaphragm<br>8. Foam/jelly<br>9. Lam<br>10. Rhythm/calendar<br>11. Other (specify)__________________	
6.20	Have you ever tested for HIV?<br>***NO⟶ End.***	1. Yes<br>2. No	

*****Thank you for your time*****

End time: ___________________

Interview result codes

1. Completed

2. Partly completed- give reason ____

3. Other (specify) ____
